# Supplementary material for: Hypomorphic SI genetic variants are associated with childhood chronic loose stools
Source: PLoS One. 2020 May 20;15(5):e0231891. doi: 10.1371/journal.pone.0231891 (PMC7239456; doi:10.1371/journal.pone.0231891)
Supplement: S1 Table — Ala, alanine; Arg, arginine; Asp, aspartate; Cys, cysteine; Gln, glutamine; Glu, glutamate; Gly, glycine; I, isomaltase; Ile, isoleucine; Leu, leucine; N/A, not available; Phe, phenylalanine Pro, proline; S, sucrase; Ser, serine; Thr, threonine; Tyr, tyrosine; Trp, tryptophan; Val, valine. (DOCX) [file pone.0231891.s001.docx]

## **Supplemental Table 1. Hypomorphic *SI* Variants Identified in Children Diagnosed with Chronic Gastrointestinal Symptoms^1-9^**

| *SI* Variants | Amino Acid Position | Protein Substitution | RS Number | Transcript Consequence | *SI* Domain | Grantham Score | PolyPhen |
| --- | --- | --- | --- | --- | --- | --- | --- |
| p.Pro348Leu | 348 | LEU, PRO | RS77546399 | c.1043C>T | I | 98 | Probable |
| p.Val577Gly | 577 | GLY,VAL | RS121912615 | c.1730T>G | I | 109 | Probable |
| p.Thr694Pro | 694 | PRO, THR | RS157364974 | c.2080A>C | I | 38 |  |
| p. Arg774Gly | 774 | GLY, ARG | RS147207752 | c.2320A>G | I | 125 | Probable |
| p.Phe875Ser | 875 | SER, PHE | Unavailable |  | I | 155 |  |
| p. Gly1073Asp | 1073 | ASP, GLY | RS121912616 | c.3218G>A | S | 94 | Probable |
| p.Gln1098Pro | 1098 | PRO, GLN | RS121912611 | c.3293A>C | S | 42 |  |
| p.Arg1124Stop | 1124 | STOP, ARG | RS200451408 |  | S | N/A | Unknown |
| p.Cys1229Tyr | 1229 | TYR, CYS | RS121912614 | c.3686G>A | S | 194 |  |
| p.Phe1745Cys | 1745 | CYS,PHE | RS79717168 | c.5234T>G | S | 205 | Probable |
| p.Trp105Cys | 105 | CYS, TRP | RS138564183 | c.315G>T | Stalk | 215 | Probable |
| p.Gln117Arg | 117 | ARG, GLN | RS121912612 | c.350A>G | I | 43 |  |
| p.Phe139Tyr | 139 | TYR, PHE | RS758407768 |  | I | 22 |  |
| p.Gln307Tyr | 307 | TYR, GLN | RS775241023 |  | I | 99 |  |
| p.Leu341Pro | 341 | PRO, LEU | RS267607049 | c.1022T>C | I | 98 |  |
| p.Asp500Glu | 500 | GLU, ASP | RS776358754 |  | I | 0 |  |
| p.Asp536Val | 536 | VAL, ASP | RS376816463 | c.1607A>T | I | 152 | Possible |
| p.Deletion545Deletion | 545 | Deletion |  |  | I |  |  |
| p.Val563Gly | 563 | GLY, VAL | RS774441965 | c.1689A>G | I | 109 |  |
| p.Ser594Pro | 594 | PRO, SER | RS765433197 | c.1780T>C | I | 74 |  |
| p.Glu613Stop | 613 | STOP, GLU | RS2011055347 |  | I |  |  |
| p.Leu620Pro | 620 | PRO, LEU | RS121912613 | c.1859T>C | I | 98 |  |
| p.Cys635Arg | 635 | ARG, CYS | RS767864714 | c.1905T>C | I | 180 |  |
| p.Leu741Pro | 741 | PRO, LEU | Unavailable |  | I | 98 |  |
| p. Arg774Lys | 774 | LYS, ARG | RS143885457 | c.2321G>A | I | 26 | Benign |
| p.Glu801Stop | 801 | STOP, GLU | RS200972419 | c.2401G>T | I | N/A | Unknown |
| p. Gln930Arg | 930 | ARG, GLN | RS150927256 | c.2789A>G | I | 43 | Benign |
| p.Trp931Arg | 931 | ARG, TRP | Unavailable |  | I |  |  |
| p.Trp931Stop | 931 | STOP, TRP | Unavailable |  | I |  |  |
| p.Arg1367Gly | 1367 | GLY, ARG | RS143388292 | c.4099A>G | S | 125 | Probable |
| p. Ile1378Ser | 1378 | SER, ILE | RS148831941 | c.4133T>G | S | 142 | Probable |
| p.Tyr1417Stop | 1417 | STOP, TYR | RS142090504 | c.4251T>G | S | N/A | Unknown |
| p.Ala1476Gly | 1476 | GLY, ALA | RS758043919 | c.4427G>C | S | 60 |  |
| p.Cys1531Tyr | 1531 | TYR, CYS | Unavailable |  | S | 194 |  |
| p.Arg1544Cys | 1544 | CYS, ARG | RS587777079 | c.4630C>T | S | 180 |  |
| p.Thr1606Ile | 1606 | ILE, THR | RS376062850 | c.4817C>T | S | 89 | Probable |
| p. Gly1760Asp | 1760 | ASP, GLY | RS145556619 | c.5279G>A | S | 94 | Benign |

**References**

**1**. Alfalah M, Keiser M, Leeb T, Zimmer KP, Naim HY. Compound heterozygous mutations affect protein folding and function in patients with congenital sucrase-isomaltase deficiency. Gastroenterology. 2009;136(3):883-92. doi: 10.1053/j.gastro.2008.11.038

**2**. Gericke B, Amiri M, Naim HY. The multiple roles of sucrase-isomaltase in the intestinal physiology. Mol Cell Pediatr. 2016;3(1):2. doi: 10.1186/s40348-016-0033-y

**3.** Jacob R, Zimmer KP, Schmitz J, Naim HY. Congenital sucrase-isomaltase deficiency arising from cleavage and secretion of a mutant form of the enzyme. J Clin Invest. 2000;106(2):281-7. doi: 10.1172/JCI9677

**4.** Keiser M, Alfalah M, Pröpsting MJ, Castelletti D, Naim HY. Altered folding, turnover, and polarized sorting act in concert to define a novel pathomechanism of congenital sucrase-isomaltase deficiency. J Biol Chem. 2006;281(20):14393-9. doi: 10.1074/jbc.M513631200

**5.** Naim HY, Heine M, Zimmer KP. Congenital sucrase-isomaltase deficiency: heterogeneity of inheritance, trafficking, and function of an intestinal enzyme complex. J Pediatr Gastroenterol Nutr. 2012;55(suppl 2):S13-20. doi: 10.1097/01.mpg.0000421402.57633.4b

**6.** Ritz V, Alfalah M, Zimmer KP, Schmitz J, Jacob R, Naim HY. Congenital sucrase-isomaltase deficiency because of an accumulation of the mutant enzyme in the endoplasmic reticulum. Gastroenterology. 2003;125(6):1678-85. doi: 10.1053/j.gastro.2003.09.022

**7.** Sander P, Alfalah M, Keiser M, Korponay-Szabo I, Kovács JB, Leeb T, et al. Novel mutations in the human sucrase-isomaltase gene (*SI*) that cause congenital carbohydrate malabsorption. Human Mutat. 2006;27(1):119**.** doi: 10.1002/humu.9392

**8**. Spodsberg N, Jacob R, Alfalah M, Zimmer KP, Naim HY. Molecular basis of aberrant apical protein transport in an intestinal enzyme disorder. J Biol Chem. 2001;276(26):23506-10. doi: 10.1074/jbc.C100219200

**9.** Uhrich S, Wu Z, Huang JY, Scott CR. Four mutations in the SI gene are responsible for the majority of clinical symptoms of CSID. J Pediatr Gastroenterol Nutr. 2012;55(suppl 2):S34-5. doi: 10.1097/01.mpg.0000421408.65257.b5
