# Supplementary material for: Hypomorphic SI genetic variants are associated with childhood chronic loose stools
Source: PLoS One. 2020 May 20;15(5):e0231891. doi: 10.1371/journal.pone.0231891 (PMC7239456; doi:10.1371/journal.pone.0231891)
Supplement: S2 Table — (DOCX) [file pone.0231891.s002.docx]

## **Supplemental Table 2. Study Questionnaire**

| **No.** | **Question** |
| --- | --- |
| **1** | What is your or your child’s primary stomach problem:  a. Stomach pain  b. Diarrhea |
| **2** | How long have you or your child been experiencing this primary stomach problem?  a. Not at all  b. 1–2 weeks  c. 3–4 weeks  d. 2–4 months  e. 5–7 months  f. 8–11 months  g. Over a year |
| **3** | On average, rate the pain you have experienced in your stomach over the last month?   |
| **4** | In the last month, how often do you or your child experience stomach pain per day?  a. Never  b. 1 time per day  c. 2 or more times per day  d. All the time (constant pain) |
| **5** | In the last month, how many days per week do you or your child experience stomach pain?  a. Never  b. One day per week  c. 2–4 days per week  d. 5–6 days per week  e. Every day |
| **6** | In the last month, how many stools have you or your child had on an average day?  1 2 3 4 5 6 7 8 9 10+ |
| **7** | In the last month, how many days per week do you or your child experience diarrhea?  a. Never  b. One day per week  c. 2–4 days per week  d. 5–6 days per week  e. Every day |
| **8** | Which picture shows you or your child’s stool when you or your child have diarrhea?   |
| **9** | In the last month, how often do you or your child experience gas per day?  a. Never  b. 1 time per day  c. 2 or more times per day  d. All the time (constant pain) |
| **10** | In the last month, how many days per week do you or your child experience gas?  a. Never  b. One day per week  c. 2–4 days per week  d. 5–6 days per week  e. Every day |
