# Supplementary material for: Hypomorphic SI genetic variants are associated with childhood chronic loose stools
Source: PLoS One. 2020 May 20;15(5):e0231891. doi: 10.1371/journal.pone.0231891 (PMC7239456; doi:10.1371/journal.pone.0231891)
Supplement: Attachment — Submitted filename: Response to Reviewers.docx [file pone.0231891.s004.docx]

*Reviewer #1: The manuscript compiles the results of a large multicenter study on the relation between functional GI symptoms in children and 37 variants of the sucrase-isomaltase gene that have been previously characterized at the biochemical and cellular levels in congenital sucrase-isomaltase deficiency (CSID). The study demonstrates a significantly higher prevalence of hypomorphic SI variants in the cases studied than in the general population.
The study supports recent views that heterozygous genotype of hypomorphic SI may be associated with an increased risk of irritable bowel syndrome. The study is properly conducted and impressive in terms of the number of cases studied. The statistical analyses are convincing and the conclusions are sound. The manuscript is clearly written (only lines 95/96 should be rewritten).
I fully agree with the conclusions, have no points of criticism and recommend the acceptance of the manuscript in its present form.*

We thank the Reviewer for his/her highly supportive comments of the manuscript. We have rewritten lines 95-96 as requested.

*Reviewer #2: The manuscript by Chumpitazi et al assesses the prevalence of variants in the sucrase-isomaltase gene among a cohort of children with chronic diarrhea. The authors find that confirmed or predicted loss of function mutations are more prevalent in the studied population versus a the EXAC reference genomic database. Furthermore, they show that patients with mutations have more diarrhea symptoms than the rest of the patient cohort. The study suggests a potentially interesting possible association between mild chronic diarrhea and SI function in young children. The strengths of the paper are that the patient cohort is taken from multiple different centers / sites and sample genotyping was done in a uniform manner at a central site. The statistics and results, while limited in scope are appropriately done.

1. The major limitation in understanding the significance of these findings, as briefly but insufficiently discussed, is that the functional effect of heterozygosity in the various loss-of-function mutations, and especially the common variants found in these patients remain poorly described and essentially unknown. The authors cite a single case report and a study on 13C breath testing in functional GI disorders (not specifically with known SI genotype), neither of which is sufficient to address the question of SI heterozygosity and enzyme activity. To assess the functional significance requires prospective clinical studies with functional measurements (enzyme/breath testing) correlated to genotype as well as basic cell-based studies assessing heterozygous vs homozygous mutations and function / localization. This should be more clearly part of the discussion and the major limitations of the existing literature (studies of symptom correlation rather than objective enzyme activity, few studies, no cellular studies etc) more straightforwardly stated.*

We thank the Reviewer for his/her supportive comments and critiques of the manuscript. We agree that the functional effect of heterozygosity for the identified mutations needs to be further elucidated. We have edited the Discussion within the manuscript to more clearly delineate the need for further investigation.

*2. In the methods the reference database link should be updated to either Gnomad or the legacy EXAC only data. There should be brief sentence explaining this update.*

We have updated the reference database link and provided an explanation for the update.
